# Quantitative Determination of Stilbenoids and Dihydroisocoumarins in *Shorea roxburghii* and Evaluation of Their Hepatoprotective Activity

**DOI:** 10.3390/ijms18020451

**Published:** 2017-02-20

**Authors:** Kiyofumi Ninomiya, Saowanee Chaipech, Yusuke Kunikata, Ryohei Yagi, Yutana Pongpiriyadacha, Osamu Muraoka, Toshio Morikawa

**Affiliations:** 1 Pharmaceutical Research and Technology Institute, Kindai University, 3-4-1 Kowakae, Higashi-osaka, Osaka 577-8502, Japan; ninomiya@phar.kindai.ac.jp (K.N.); chaipechann@hotmail.com (S.C.); yusuke.kunikata@takeda.com (Y.K.); poseidaon.acchi-kocchi-docchi@docomo.ne.jp (R.Y.); muraoka@phar.kindai.ac.jp (O.M.); 2 Antiaging Center, Kindai University, 3-4-1 Kowakae, Higashi-osaka, Osaka 577-8502, Japan; 3 Faculty of Agro-Industry, Rajamangala University of Technology Srivijaya, Thungyai, Nakhon Si Thammarat 80240, Thailand; 4 Faculty of Science and Technology, Rajamangala University of Technology Srivijaya, Thungyai, Nakhon Si Thammarat 80240, Thailand; yutanap@hotmail.com

**Keywords:** *Shorea roxburghii*, stilbenoid, dihydroisocoumarin, quantitative analysis, hepatoprotective effect, mechanism of action

## Abstract

A simultaneous quantitative analytical method for 13 stilbenoids including (−)-hopeaphenol (**1**), (+)-isohopeaphenol (**2**), hemsleyanol D (**3**), (−)-ampelopsin H (**4**), vaticanols A (**5**), E (**6**), and G (**7**), (+)-α-viniferin (**8**), pauciflorol A (**9**), hopeafuran (**10**), (−)-balanocarpol (**11**), (−)-ampelopsin A (**12**), and *trans*-resveratrol 10-*C*-β-d-glucopyranoside (**13**), and two dihydroisocoumarins, phayomphenols A_1_ (**14**) and A_2_ (**15**) in the extract of *Shorea roxburghii* (dipterocarpaceae) was developed. According to the established protocol, distributions of these 15 polyphenols (**1**–**15**) in the bark and wood parts of *S. roxburghii* and a related plant *Cotylelobium melanoxylon* were evaluated. In addition, the principal polyphenols (**1**, **2**, **8**, **13**–**15**) exhibited hepatoprotective effects against d-galactosamine (d-galN)/lipopolysaccharide (LPS)-induced liver injury in mice at a dose of 100 or 200 mg/kg, p.o. To characterize the mechanisms of action, the isolates were examined in in vitro studies assessing their effects on (i) d-GalN-induced cytotoxicity in primary cultured mouse hepatocytes; (ii) LPS-induced nitric oxide (NO) production in mouse peritoneal macrophages; and (iii) tumor necrosis factor-α (TNF-α)-induced cytotoxicity in L929 cells. The mechanisms of action of these polyphenols (**1**, **2**, and **8**) were suggested to be dependent on the inhibition of LPS-induced macrophage activation and reduction of sensitivity of hepatocytes to TNF-α. However, none of the isolates reduced the cytotoxicity caused by d-GalN.

## 1. Introduction

**Figure 1:**
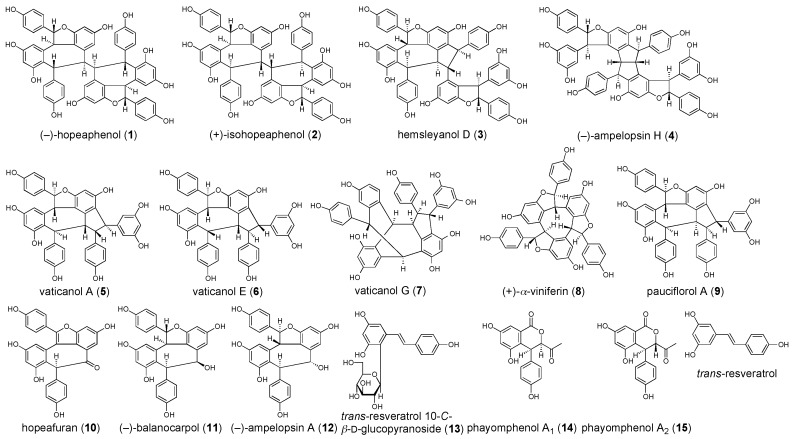
Stilbenoids and dihydroisocoumarins (**1**–**15**) from the bark of *Shorea roxburghii*.

Stilbenoids, a family of polyphenols, are known for the complexity of their structure and diverse biological activities [1]. They occur with a limited but heterogeneous distribution in the plant kingdom. Some phylogenetically distant botanical families (e.g., Gnetaceae, Pinaceae, Cyperaceae, Fabaceae, Dipterocarpaceae, and Vitaceae) are well recognized as rich sources of stilbenoids and their oligomers (oligostilbenoids) [1]. *Trans*-resveratrol (3,5,4′-trihydroxy-*trans*-stilbene), one of the most popular naturally occurring stilbenoids, has been reported to have anti-aging properties as well as beneficial health effects in patients with cancer, cardiovascular, inflammatory, and central nervous system diseases [2,3,4,5,6,7]. The majority of the oligostilbenoids are regioselectively synthesized by phenoxy radical coupling of resveratrol [8,9,10,11,12]. A Dipterocarpaceae plant *Shorea roxburghii* G. Don is widely distributed in Thailand and its neighboring countries such as Cambodia, India, Laos, Malaysia, Myanmar, and Vietnam. In Thailand, this plant is locally called “Phayom”, and its bark has been used as an astringent and preservative for traditional beverages. In the course of our studies on bioactive constituents from *S. roxburghii*, we have isolated the following 13 stilbenoids, (−)-hopeaphenol (**1**), (+)-isohopeaphenol (**2**), hemsleyanol D (**3**), (−)-ampelopsin H (**4**), vaticanols A (**5**), E (**6**), and G (**7**), (+)-α-viniferin (**8**), pauciflorol A (**9**), hopeafuran (**10**), (−)-balanocarpol (**11**), (−)-ampelopsin A (**12**), and *trans*-resveratrol 10-*C*-β-d-glucopyranoside (**13**), and two dihydroisocoumarins, phayomphenols A_1_ (**14**) and A_2_ (**15**), from methanol extract of the bark part as they were present in relative abundance (Figure 1) [13,14]. We also revealed that the methanol extract and its constituents displayed anti-hyperlipidemic and anti-diabetogenic effects in olive-oil and sucrose-loaded mice, respectively. These effects were noted in their corresponding target enzymatic inhibitory activities, such as pancreatic lipase, small intestinal α-glucosidase, and lens aldose reductase [13,14]. Furthermore, we have recently reported that several oligostilbenoids (**1**, **3**, and **8**) possess more potent anti-proliferative properties than that of a corresponding monomer, *trans*-resveratrol, against SK-MEL-28 human malignant melanoma cells [15]. Thus, the plant *S. roxburghii* is considered a promising abundant resource for these bioactive oligostilbenoids. In this paper, we propose a simple, rapid, and precise analytical method for high performance liguid chromatography (HPLC) simultaneous quantitative determination of 13 stilbenoids (**1**–**13**) and two dihydroisocoumarins (**14** and **15**) in *S. roxburghii* using a one-step sample preparation procedure. In addition, we also describe the hepatoprotective effects of the principal isolates from the bark of *S. roxburghii* as well as their possible mechanisms of action.

## 2. Results and Discussion

### 2.1. Isolation

Studies have shown that the principal compounds (**1**–**15**) as well as following compounds—vaticanols B and C, malibatols A and B, (+)-parviflorol, *cis*-resveratrol 10-*C*-β-d-glucopyranoside, *trans*-piceid, and 1′*S*-dihydrophayomphenol A_2_ (Figure S1)—were obtained from the bark of *S. roxburghii* [13,14].

### 2.2. Simultaneous Quantitative Analysis

**Figure 2:**
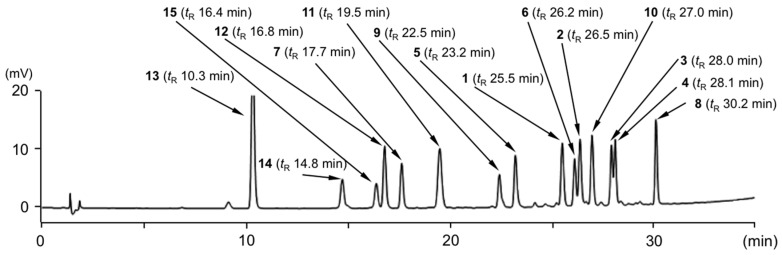
Typical HPLC chromatogram (UV, 284 nm) of standard solution mixture (each 25 µg/mL).

As shown in Figure 2, a typical HPLC chromatogram for a standard solution mixture (**1**–**15**) under UV detection (284 nm) demonstrated good baseline separation for all peaks. Each peak was observed at the following retention time (*t*_R_): **1**: 25.5 min; **2**: 26.5 min; **3**: 28.0 min; **4**: 28.1 min; **5**: 23.2 min; **6**: 26.2 min; **7**: 17.7 min; **8**: 30.2 min; **9**: 22.5 min; **10**: 27.0 min; **11**: 19.5 min; **12**: 16.8 min; **13**: 10.3 min; **14**: 14.8 min; **15**: 16.4 min. These peaks were unambiguously assigned by comparing of their retention times with those of authentic specimens.

In order to optimize the extraction condition, the quality of the extracts in association with the contents of the stilbenoids (**1**–**13**) and dihydroisocoumarins (**14** and **15**) were examined. The extraction efficacies were compared for three solvent systems (methanol, 50% aqueous methanol, and water) under two different conditions (reflux for 120 min or sonication for 30 min, each twice). As shown in Table 1, “reflux in methanol” afforded the highest total contents of these polyphenols (**1**–**15**). Therefore, all of the analytical samples were prepared using the method “reflux in methanol for 120 min, twice”. As shown in Table 2, analytical parameters such as linearity, limits of detection and quantitation, and precision of the developed method were evaluated. The calibration curves were linear in the range studied (2.5–50 µg/mL), showing the correlation coefficients (*R*^2^) of more than 0.9994 for each analyte. Linear regression equations of their calibration curves for each analyte were described in Table 2. The detection and quantitation limits were estimated to be 0.05–0.26 and 0.14–0.80 ng, respectively, indicating sufficient sensitivity of this method. The relative standard deviation (RSD) values were 0.13%–1.54% for intra-day assays and 0.13%–1.58% for inter-day assays. Accuracy was determined in recovery experiments using the methanol under reflux extract from the bark of *S. roxburghii*. As shown in Table 3, overall recovery rate was observed in the range of 95.1%–104.9% with RSD values lower than 1.3%. According to the established protocol, contents of the stilbenoids (**1**–**13**) and dihydroisocoumarins (**14** and **15**) in both bark and wood of *S. roxburghii*, as well as a related plant classified the same Dipterocarpaceae family *Cotylelobium melanoxylon*, which have all been reported to possess the common oligostilbenoids, were evaluated [16] (Figure S2). The assays proved to be reproducible, precise, and readily applicable to the quality evaluation of these extracts. As shown in Table 4, total polyphenol content in the bark of *S. roxburghii* (72.60 mg/g in dry material) was found to be three-fold higher than the wood part (21.20 mg/g). Among them, two resveratrol tetramers, (−)-hopeaphenol (**1**, 13.31 mg/g in dry material) and (+)-isohopeaphenol (**2**, 10.21 mg/g), a resveratrol trimer, vaticanol E (**6**, 11.57 mg/g), and a dihydroisocoumarin, phayomphenol A_1_ (**13**, 13.81 mg/g) were present relatively in abundance in the bark of *S. roxburghii*. As for *C. melanoxylon*, both of the total oligostilbenoid contents in the bark (286.73 mg/g) and wood (197.50 mg/g) were higher than those of *S. roxburghii*, and their distributions were biased towards vaticanols A (**5**, 76.45 mg/g in the bark), E (**6**, 120.75 mg/g in the bark), and G (**7**, 63.81 mg/g in the bark; 181.69 mg/g in the wood), which is supported by our previous report [16]. On the basis of this evidence, these Dipterocarpaceaeous plants, *S. roxburghii* and *C. melanoxylon*, have been shown to be useful as abundant resources for obtaining the bioactive oligostilbenoids.

### 2.3. Protective Effects of Principal Polyphenols (**1**, **2**, **8**, and **13**–**15**) on Liver Injury Induced by d-GalN/LPS in Mice

Infection with hepatitis C virus and chronic consumption of alcohol are major causes of liver injury, cirrhosis, and hepatocellular carcinoma worldwide. Tumor necrosis factor-α (TNF-α) is known to mediate organ injuries through its induction of cellular inflammatory responses. In the liver, the biological effects of TNF-α have been implicated in hepatic injuries associated with hepatic toxins, ischemia/reperfusion, viral hepatitis, and alcoholic liver disease or alcohol-related disorders [17,18,19]. Therefore, TNF-α is considered as an important target in the attempt to discover anti-inflammatory and hepatoprotective agents. The d-GalN/lipopolysaccharide (LPS)-induced liver injury model is recognized to develop via immunological responses [20]. This model causes liver injury in two steps. First, expression of inhibitors of apoptosis proteins (IAPs) is inhibited by administration of d-GalN through depletion of uridine triphosphate in hepatocytes. Second, pro-inflammatory mediators such as nitric oxide (NO), reactive oxygen species (ROS), and TNF-α are released from LPS-activated macrophages (Kupffer’s cells). Apoptosis of hepatocytes induced by TNF-α plays an important role in d-GalN/LPS-induced liver injury [21]. In our previous studies on hepatoprotective properties of compounds obtained from natural resources, we have already reported that sesquiterpenes [22,23,24,25], triterpenes [26], limonoids [27], coumarins [28], acid amides [29,30,31], phenylethanoids [32], and saponins [33] exhibited significant protective effects against liver injuries induced by d-GalN/LPS in mice. In the present study, we investigated the protective effects of principal polyphenols, (−)-hopeaphenol (**1**), (+)-isohopeaphenol (**2**), (+)-α-viniferin (**8**), *trans*-resveratrol 10-*C*-β-d-glucopyranoside (**13**), and phayomphenols A_1_ (**14**) and A_2_ (**15**), on the d-GalN/LPS-induced liver injury. As shown in Table 5, all of the tested compounds (**1**, **2**, **8**, and **13**–**15**) at a dose of 100 or 200 mg/kg, p.o. significantly inhibited the increase in serum levels of aspartate amino transaminase (sAST) and alanine amino transaminase (sALT), which served as markers of acute liver injury [34,35,36]. A corresponding stilbene monomer resveratrol has been reported to ameliorate hepatotoxicity in several in vivo liver injury models, such as streptozotocin-induced diabetic [37], acetoaminiphen-induced [38], and ethanol-induced oxidative stress models [39]. The hepatoprotective activities of compounds **1**, **2**, **8**, and **13**–**15** in this model were equivalent to or more potent than *trans*-resveratrol.

### 2.4. Effects on d-GalN-Induced Cytotoxicity in Primary Cultured Mouse Hepatocytes

To characterize the mechanisms responsible for the hepatoprotective activity, the inhibitory effect of 23 polyphenol constituents, including principal polyphenols (**1**–**15**) isolated from the bark of *S. roxburghii* [13,14], on d-GalN-induced cytotoxicity in primary cultured mouse hepatocytes were examined. As shown in Table 6, none of the isolates led to a reduction in the cytotoxicity at concentrations of up to 100 μM. These results are similar to what was seen with curcumin [22,23,25,27], a naturally occurring hepatoprotective product obtained from turmeric [40]. Thus, the principal polyphenols (**1**–**15**) did not affect d-GalN-induced cytotoxicity. In contrast, *trans*-resveratrol inhibited the cytotoxicity (IC_50_ = 40.8 μM), which was equivalent to that of silybin (IC_50_ = 38.8 μM) [26,27,30,32], a naturally occurring hepatoprotective product obtained from milk thistle [41,42]. This evidence led us to confirm that *trans*-resveratrol and its oligomers do not have similar effects on d-GalN-induced cytotoxicity in hepatocytes.

### 2.5. Effects on LPS-Induced NO Production in Mouse Peritoneal Macrophages

The effects of the polyphenol constituents from the bark of *S. roxburghii* on NO production were examined to provide an estimation of macrophage activation levels in LPS-treated mouse peritoneal macrophages. As shown in Table 7, resveratrol tetramers, (−)-hopeaphenol (**1**, IC_50_ = 4.6 μM), (+)-isohopeaphenol (**2**, 38.5 μM), (−)-ampelopsin H (**4**, 18.6 μM), and vaticanols B (26.8 μM) and C (14.5 μM), the trimers, (+)-α-viniferin (**8**, 9.7 μM) and pauciflorol A (**9**, 17.8 μM), the dimers, hopeafuran (**10**, 45.9 μM), and malibatols A (23.0 μM) and B (18.5 μM) significantly inhibited NO production without notable cytotoxic effects at the effective concentration. The potencies of the aforementioned oligostilbenoids were higher than those of the NO synthase inhibitor, *N*^G^-monomethyl-l-arginine (l-NMMA, IC_50_ = 36.0 µM). The inhibitory activity of *trans*-resveratrol (17.8 μM) was equivalent to that of caffeic acid phenethyl ester (CAPE, 11.0 µM), an inhibitor of nuclear factor-κB activation.

### 2.6. Effects on TNF-α-Induced Cytotoxicity in L929 Cells

The effects of the isolates on the sensitivity of hepatocytes to TNF-α were assessed by measuring TNF-α-induced decreases in the viability of L929 cells, a TNF-α-sensitive cell line, using the MTT assay [27]. As shown in Table 8, oligostilbenoids such as vaticanol G (**7**, IC_50_ = 86.6 µM), (+)-α-viniferin (**8**, 15.0 µM), pauciflorol A (**9**, 26.7 µM), hopeafuran (**10**, 22.0 µM), and malibatols A (12.3 µM) and B (10.2 µM) inhibited the decrease in cell viability to a greater degree than that of silybin (IC_50_ = 60.4 µM).

## 3. Materials and Methods

### 3.1. Chemicals and Reagents

Methanol, acetic acid, and distilled water for HPLC were purchased from Nacalai Tesque Inc., Kyoto, Japan. All other chemicals were reagent grade, and were purchased from Wako Pure Chemical Industries, Ltd., Tokyo, Japan or Nacalai Tesque Inc., Kyoto, Japan.

### 3.2. Plant Materials

The bark and wood parts of *Shorea roxburghii* and *Cotylelobium melanoxylon* were collected from Phatthalung Province, Thailand, in September 2006 or September 2007 as described previously [13,16]. All of the plant materials were identified by one of the authors, Yutana Pongpiriyadacha. Voucher specimens (2006.09. Raj-02, 2006.09. Raj-02#, 2007.09. Raj-04, and 2006.09. Raj-04#, respectively) were deposited in the Garden of Medicinal Plants, Kindai University. The materials were air-dried in a shaded room for more than a month.

### 3.3. Standard Solution Preparation

An accurately weighed 20.0 mg of each compound (**1**–**15**) was introduced into a 20 mL volumetric flask, and methanol was added to make up the volume of the stock standard solution (1000 µg/mL). Aliquots of 50, 100, 250, and 500 µL of the stock standard solution were transferred into 10 mL volumetric flasks and the volume was made up with 50% aqueous methanol for use as working solutions (5.0, 10, 25, and 50 µg/mL, respectively) for constructing calibration curves. For calibration, an aliquot of 2 µL of each solution was injected into the HPLC system. Each peak was observed at the following retention times: **1** (*t*_R_ 25.5 min), **2** (*t*_R_ 26.5 min), **3** (*t*_R_ 28.0 min), **4** (*t*_R_ 28.1 min), **5** (*t*_R_ 23.2 min), **6** (*t*_R_ 26.2 min), **7** (*t*_R_ 17.7 min), **8** (*t*_R_ 30.2 min), **9** (*t*_R_ 22.5 min), **10** (*t*_R_ 27.0 min), **11** (*t*_R_ 19.5 min), **12** (*t*_R_ 16.8 min), **13** (*t*_R_ 10.3 min), **14** (*t*_R_ 14.8 min), and **15** (*t*_R_ 16.4 min).

### 3.4. Sample Solution Preparation

An accurately weighed pulverized sample powder (ca. 2 g, conversion with loss on drying) was extracted with 20 mL of three solvent systems (methanol, 50% aqueous methanol, or water) under two conditions (reflux for 120 min or sonication for 30 min, each twice). The extracts were combined and centrifuged at 3000 rpm for 5 min, then the supernatants were diluted to 100 mL with the extraction solvent. An aliquot (1 mL) of the extract solution was transferred into a 10 mL volumetric flask and 50% aqueous methanol was added to make up the volume. The solution was filtered through a syringe filter (0.45 µm), and an aliquot of 2 µL was subjected to the HPLC analysis. To calculate the extraction yields, the remaining extraction solution (90 mL) was evaporated in vacuo (Table 1).

### 3.5. HPLC Instruments and Conditions

All analytical experiments in this study were performed with an LC-20A series Prominence HPLC system (Shimadzu Co., Kyoto, Japan), which consists of a UV-VIS detector, a binary pump, a degasser, an autosampler, a thermostated column compartment, and a control module. The chromatographic separation was performed on an Inertsil ODS-3 column (3 μm particle size, 2.1 mm i.d. × 100 mm, GL Sciences Inc., Tokyo, Japan) operated at 40 °C with mobile phase A (acetonitrile) and B (H_2_O containing 0.1% formic acid). The gradient program was as follows: 0 min (A:B = 10:90, *v*/*v*) → 20 min (30:70, *v*/*v*) → 30–40 min (50:50, *v*/*v*, hold). The flow rate was 0.2 mL/min with UV detection at 284 nm and the injection volume was 2 μL.

### 3.6. Calibration and Validation

The standard curves were prepared with four concentration levels in the range of 0.5–50 µg/mL. Standard curves were made on each day of analysis. Linearity for each compound was plotted using linear regression of the peak area versus concentration. The coefficient of correlation (*R*^2^) was used to judge the linearity. The detection and quantitation limits for each analyte were determined by the signal-to-noise (*S/N*) ratio for each analyte by analyzing a series of diluted standard solutions until the *S/N* ratios were about 3 and 10, respectively, based on a 2 µL injection. Precision and accuracy of the analytical method were tested using a methanol under reflux extract of the bark of *S. roxburghii*. The intra- and inter-day precisions were determined by estimating the corresponding responses five times on the same day and on five different days, respectively (Table 2). The recovery rates were determined by adding analytes of two different concentrations (50 and 125 µg/mL) to the sample solution of the homogeneous extract (Table 3).

### 3.7. Reagents for Bioassays

LPS (from *Salmonella enteritidis*), minimum essential medium (MEM), and William’s E medium were purchased from Sigma-Aldrich Chemical (St. Louis, MO, USA); fetal bovine serum (FBS) was from Life Technologies (Rockville, MD, USA); other chemicals were from Nakalai Tesque Inc. (Kyoto, Japan) or Wako Pure Chemical Industries, Co., Ltd. (Osaka, Japan). The 96-well microplates were purchased from Sumitomo Bakelite Co., Ltd. (Tokyo, Japan).

### 3.8. Animals

Male ddY mice were purchased from Kiwa Laboratory Animal Co., Ltd. (Wakayama, Japan). The animals were housed at a constant temperature of 23 ± 2 °C and fed a standard laboratory chow (MF, Oriental Yeast Co., Ltd., Tokyo, Japan). All experiments were performed with conscious mice unless otherwise noted. The experimental protocol was approved by Kindai University’s Committee for the Care and Use of Laboratory Animals (KAPR-26-001, 1 April 2014).

### 3.9. Effects on d-GalN/LPS-Induced Liver Injury in Mice

Protective effects on d-GalN/LPS-induced liver injury in mice were determined according to the previously described protocol [27]. *Trans*-resveratrol, curcumin, and silybin were used as reference compounds.

### 3.10. Effects on Cytotoxicity Induced by d-GalN in Primary Cultured Mouse Hepatocytes

Hepatoprotective effects on d-GalN-induced cytotoxicity in primary cultured mouse hepatocytes were determined according to the protocol described previously [27]. *Trans*-resveratrol, curcumin, and silybin were used as reference compounds.

### 3.11. Effects on Production of NO in LPS-Induced Mouse Peritoneal Macrophages

Assays for NO production in TGC-induced mouse peritoneal macrophages were performed as described previously [27]. *Trans*-resveratrol, *N*^G^-Monomethyl-l-arginine (l-NMMA), and caffeic acid phenethyl ester (CAPE) were used as reference compounds.

### 3.12. Effects on Cytotoxicity Induced by TNF-α in L929 Cells

Inhibitory effects on TNF-α-induced cytotoxicity in L929 cells were determined according to the protocol described previously [27]. *Trans*-resveratrol and silybin were used as reference compounds.

### 3.13. Statistics

Values are expressed as means ± SEM. One-way analysis of variance (ANOVA) followed by Dunnett’s test was used for statistical analysis. Probability (*p*) values less than 0.05 were considered significant.

## 4. Conclusions

**Figure 3:**
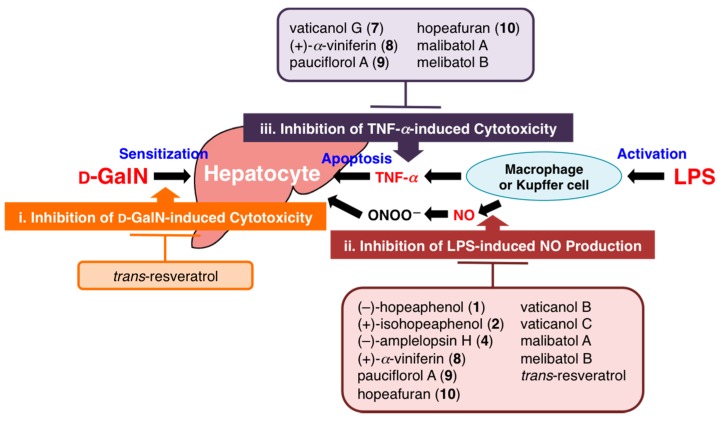
Plausible mechanisms of action of stilbenoids on d-GalN/LPS-induced liver injury.

We have developed a practical method for the simultaneous quantitative determination of 13 stilbenoids, (−)-hopeaphenol (**1**), (+)-isohopeaphenol (**2**), hemsleyanol D (**3**), (−)-ampelopsin H (**4**), vaticanols A (**5**), E (**6**), and G (**7**), (+)-α-viniferin (**8**), pauciflorol A (**9**), hopeafuran (**10**), (−)-balanocarpol (**11**), (−)-ampelopsin A (**12**), and *trans*-resveratrol 10-*C*-β-d-glucopyranoside (**13**), and two dihydroisocoumarins, phayomphenols A_1_ (**14**) and A_2_ (**15**), in the bark and wood parts of *Shorea roxburghii* and *Cotylelobium melanoxylon*. The method was validated with respect to linearity, detection limit, precision, and accuracy. The assay was reproducible and precise, and could be useful to obtain abundant resources of the bioactive oligostilbenoids. Among the isolates from the bark of *S. roxburghii*, the principal polyphenols (**1**, **2**, **8**, and **13**–**15**) exhibited protective effects against liver injury induced by d-GalN/LPS in mice at a dose of 100 or 200 mg/kg, p.o. The mechanisms of action are likely dependent on inhibition of LPS-induced macrophage activation and a reduction in sensitivity of hepatocytes to TNF-α. They did not affect the reduction of cytotoxicity caused by d-GalN, as summarized in Figure 3. It is well known that activation of NF-κB is a key factor in both activation of macrophages and TNF-α-induced cell death. The detailed mechanisms of action for the hepatoprotective effects, including the influence on NF-κB activation, require further study.

## Figures and Tables

**Table 1:** Extraction efficiency of stilbenoids (**1**–**13**) and dihydroisocoumarins (**14** and **15**) from the bark of *Shorea roxburghii*.

Extraction Method	Extraction Yield (%)	Contents (mg/g in Dry Material)															Total ^a^
		1	2	3	4	5	6	7	8	9	10	11	12	13	14	15	
Methanol, reflux	17.74	13.31	10.21	7.89	2.41	4.84	11.57	1.25	2.35	0.91	0.52	1.71	0.34	13.81	1.03	0.45	72.60 (100)
50% Methanol, reflux	20.21	13.31	10.17	7.90	2.39	4.85	11.56	1.26	2.23	0.92	0.52	1.71	0.31	14.01	1.01	0.39	72.54 (100)
H_2_O, reflux	12.28	4.72	4.64	1.62	0.57	1.65	3.51	0.65	0.15	0.37	0.17	0.69	0.16	9.66	0.67	0.36	29.59 (41)
Methanol, sonication	13.47	11.46	8.49	6.81	2.00	4.08	9.93	1.04	1.68	0.80	0.43	1.35	0.25	11.00	0.71	0.36	60.39 (83)
50% Methanol, sonication	11.67	8.42	6.47	4.83	1.49	3.06	6.18	0.85	1.12	0.61	0.36	1.00	0.20	10.95	0.65	0.31	46.50 (64)
H_2_O, sonication	12.71	6.51	6.11	2.07	0.74	2.23	4.93	0.89	n.d. ^b^	0.70	0.18	0.80	0.16	10.45	0.63	0.32	36.72 (51)

Extraction efficiency was tested using the bark of *Shorea roxburghii* (loss of drying 7.44%); ^a^ value (%) relative to the content obtained by methanol under reflux is given in parenthesis; and ^b^ less than the quantitation limit.

**Table 2:** Linearities, detection and quantitation limits, and precisions for stilbenoids (**1**–**13**) and dihydroisocoumarins (**14** and **15**) from the bark of *Shorea roxburghii*.

Analyte	Regression Equation ^a^	Correlation Coefficient	Detection Limit ^b^ (ng)	Quantitation Limit ^b^ (ng)	Precision ^c^ (RSD, %)	
					Intra-Day	Inter-Day
(−)-Hopeaphenol (**1**)	*y* = 4404 *x* − 822	0.9997	0.11	0.34	0.30	0.28
(+)-Isohopeaphenol (**2**)	*y* = 4465 *x* − 850	0.9998	0.11	0.32	0.90	1.08
Hemsleyanol D (**3**)	*y* = 3506 *x* − 923	0.9999	0.11	0.34	1.09	0.81
(−)-Ampelopsin H (**4**)	*y* = 3743 *x* − 885	0.9998	0.11	0.34	0.80	0.78
Vaticanol A (**5**)	*y* = 3747 *x* − 507	1.000	0.14	0.42	1.32	0.13
Vaticanol E (**6**)	*y* = 3009 *x* − 765	0.9994	0.14	0.44	0.73	0.91
Vaticanol G (**7**)	*y* = 3195 *x* − 487	1.000	0.14	0.44	0.49	1.25
(+)-α-Viniferin (**8**)	*y* = 4585 *x* − 166	0.9999	0.08	0.26	1.54	1.23
Pauciflorol A (**9**)	*y* = 2592 *x* − 441	1.000	0.22	0.66	0.46	0.78
Hopeafuran (**10**)	*y* = 4518 *x* − 150	1.000	0.10	0.30	0.84	1.29
(−)-Balanocarpol (**11**)	*y* = 5666 *x* + 325	0.9999	0.12	0.36	0.46	1.58
(−)-Ampelopsin (**12**)	*y* = 4516 *x* − 427	1.000	0.10	0.32	0.35	0.32
*Trans*-resveratrol 10-*C*-Glc (**13**)	*y* = 11461 *x* − 872	1.000	0.05	0.14	0.13	0.96
Phayomphenol A_1_ (**14**)	*y* = 2700 *x* − 564	0.9999	0.24	0.72	0.93	0.60
Phayomphenol A_2_ (**15**)	*y* = 2059 *x* − 189	0.9999	0.26	0.80	0.36	0.93

^a^ In the regression equation, *x* is the concentration of the analyte solution (µg/mL), and *y* is the peak area of the analyte; ^b^ values are the amount of the analyte injected on-column; and ^c^ precision of the analytical method were tested using the methanol under reflux extract of the bark of *Shorea roxburghii* (*n* = 5).

**Table 3:** Recoveries for stilbenoids (**1**–**13**) and dihydroisocoumarins (**14** and **15**) from the bark of *Shorea roxburghii*.

Add (µg/mL)	Recovery ^a^ (%)														
	1	2	3	4	5	6	7	8	9	10	11	12	13	14	15
50	96.1 ± 1.0	97.1 ± 1.1	95.1 ± 1.1	101.1 ± 1.0	98.0 ± 0.9	95.8 ± 0.7	99.8 ± 0.9	100.6 ± 1.1	104.9 ± 1.2	102.7 ± 1.0	96.2 ± 1.3	98.8 ± 0.8	95.7 ± 0.9	98.1 ± 0.5	98.3 ± 1.0
125	104.9 ± 0.9	103.1 ± 1.2	101.6 ± 0.6	100.7 ± 1.0	99.0 ± 1.1	100.7 ± 1.0	96.3 ± 1.1	95.9 ± 1.0	104.3 ± 1.0	103.7 ± 1.2	101.5 ± 0.9	96.5 ± 0.5	101.2 ± 0.5	102.4 ± 0.6	99.4 ± 0.3

^a^ The recovery rates were determined by adding analytes of two different concentrations (50 and 125 µg/mL) to the sample solution; recoveries spiked with the methanol under reflux extract of the bark of *S. roxburghii* (*n* = 3).

**Table 4:** Contents of stilbenoids (**1**–**13**) and dihydroisocoumarins (**14** and **15**) in the extracts from the bark or wood of *Shorea roxburghii* and *Cotylelobium melanoxylon*.

Extraction Method	Loss of Drying ^a^ (%)	Extraction Yield ^b^ (%)	Contents (mg/g in Dry Material)															Total
			1	2	3	4	5	6	7	8	9	10	11	12	13	14	15	
*S. roxburghii*, bark	7.44	17.74	13.31	10.21	7.89	2.41	4.84	11.57	1.25	2.35	0.91	0.52	1.71	0.34	13.81	1.03	0.45	72.60
*S. roxburghii*, wood	6.76	8.32	3.39	3.03	1.83	0.51	2.04	3.75	0.40	n.d. ^c^	n.d. ^c^	n.d. ^c^	2.03	0.28	3.94	n.d. ^c^	n.d. ^c^	21.20
*C. melanoxylon*, bark	8.96	31.40	n.d. ^c^	n.d. ^c^	24.59	n.d. ^c^	76.45	120.75	63.81	n.d. ^c^	1.13	n.d. ^c^	n.d. ^c^	n.d. ^c^	n.d. ^c^	n.d. ^c^	n.d. ^c^	286.73
*C. melanoxylon*, wood	7.77	27.89	n.d. ^c^	n.d. ^c^	4.91	n.d. ^c^	7.45	3.45	181.69	n.d. ^c^	n.d. ^c^	n.d. ^c^	n.d. ^c^	n.d. ^c^	n.d. ^c^	n.d. ^c^	n.d. ^c^	197.50

^a^ Each powdered sample was dried 105 °C for 8 h; ^b^ each powdered sample was extracted two times with methanol under reflux for 120 min; and ^c^ less than the quantitation limit.

**Table 5:** Inhibitory effects of principal polyphenol constituents (**1**, **2**, **8**, and **13**—**15**) on d-GalN/lipopolysaccharide (LPS)-induced liver injury in mice.

Treatment	Dose (mg/kg, p.o.)	*n*	Inhibition (%)	
			sAST	sALT
(−)-Hopeaphenol (**1**)	100	6	92.2 ± 5.4 **	90.6 ± 6.5 **
(+)-Isohopeaphenol (**2**)	100	6	80.6 ± 4.1 **	79.8 ± 3.6 **
(+)-α-Viniferin (**8**)	100	6	70.1 ± 4.0 **	69.5 ± 6.2 **
–	200	6	75.3 ± 5.5 **	71.9 ± 2.6 **
*Trans*-resveratrol 10-*C*-Glc (**13**)	100	7	47.8 ± 4.8	43.9 ± 11.8
–	200	7	65.7 ± 3.4 **	67.5 ± 12.3 **
Phayomphenol A_1_ (**14**)	100	7	48.0 ± 6.0 *	47.0 ± 6.5 *
Phayomphenol A_2_ (**15**)	100	7	61.0 ± 8.1 **	64.7 ± 6.9 **
*Trans*-resveratrol	100	7	52.0 ± 9.0 *	51.2 ± 7.9 *
–	200	5	55.7 ± 9.9 *	63.1 ± 5.0 **
Curcumin [26,27]	12.5	10	21.1 ± 20.0	24.0 ± 2.6
–	25	10	47.8 ± 16.0	50.9 ± 14.6
–	50	9	63.8 ± 9.1 *	71.2 ± 7.1 *
Silybin [27]	500	8	71.1 ± 6.8 **	71.9 ± 3.1 **

Each value represents the mean ± SEM; asterisks denote significant differences from the control group, * *p* < 0.05, ** *p* < 0.01; commercial resveratrol was purchased from Wako Pure Chemical Industries, Ltd. (Osaka, Japan), whereas silybin was from Funakoshi Co., Ltd. (Tokyo, Japan).

**Table 6:** Inhibitory effects of constituents from the bark of *Shorea roxburghii* on d-GalN-induced cytotoxicity in mouse primary hepatocytes.

Treatment	Inhibition (%)				
	0 µM	3 µM	10 µM	30 µM	100 µM
(−)-Hopeaphenol (**1**)	0.0 ± 0.6	−5.8 ± 1.6	−4.5 ± 1.3	−5.0 ± 0.3	−5.8 ± 1.5
(+)-Isohopeaphenol (**2**)	0.0 ± 1.4	−4.9 ± 0.4	−7.9 ± 1.2	−10.2 ± 0.4	−12.5 ± 0.9
Hemsleyanol D (**3**)	0.0 ± 1.3	−7.2 ± 0.5	−12.1 ± 0.5	−7.8 ± 0.9	−23.2 ± 0.5
(−)-Ampelopsin H (**4**)	0.0 ± 0.2	−7.1 ± 0.7	−7.1 ± 1.2	−10.5 ± 1.3	−17.3 ± 0.3
Vaticanol A (**5**)	0.0 ± 0.9	1.6 ± 0.5	−1.3 ± 0.5	2.2 ± 1.1	−2.0 ± 1.1
Vaticanol E (**6**)	0.0 ± 0.4	−3.1 ± 0.7	−3.0 ± 1.0	−2.4 ± 0.6	−0.3 ± 1.0
Vaticanol G (**7**)	0.0 ± 0.6	−7.5 ± 0.7	−7.0 ± 1.7	−6.6 ± 1.7	−12.7 ± 1.0
(+)-α-Viniferin (**8**)	0.0 ± 2.1	−3.3 ± 1.9	10.1 ± 2.1	32.4 ± 4.0 **	−29.9 ± 0.6
Pauciflorol A (**9**)	0.0 ± 1.4	−1.5 ± 0.9	−5.0 ± 0.7	−6.1 ± 0.4	−10.2 ± 0.4
Hopeafuran (**10**)	0.0 ± 1.4	−6.7 ± 0.4	0.8 ± 1.4	13.0 ± 0.9 **	−22.0 ± 0.6
(−)-Balanocarpol (**11**)	0.0 ± 1.5	−6.4 ± 0.3	−3.1 ± 2.0	11.8 ± 2.7 **	13.5 ± 0.8 **
(−)-Ampelopsin A (**12**)	0.0 ± 1.8	−2.9 ± 0.8	−1.4 ± 1.2	22.5 ± 1.5 **	29.4 ± 1.0 **
*Trans*-resveratrol 10-*C*-Glc (**13**)	0.0 ± 2.0	7.1 ± 1.9	15.4 ± 2.5 **	12.8 ± 2.3 **	2.5 ± 0.8
Phayomphenol A_1_ (**14**)	0.0 ± 1.3	5.1 ± 6.8	12.2 ± 4.8	26.9 ± 3.2 **	42.7 ± 4.3 **
Phayomphenol A_2_ (**15**)	0.0 ± 2.2	1.7 ± 2.8	13.0 ± 1.9 *	16.1 ± 3.0 **	33.9 ± 3.0 **
Vaticanol B	0.0 ± 1.4	−1.5 ± 0.9	−5.0 ± 0.7	−6.1 ± 0.4	−10.2 ± 0.4
Vaticanol C	0.0 ± 2.1	−5.7 ± 2.0	−4.3 ± 1.5	−5.6 ± 2.2	−12.0 ± 1.3
Malibatol A	0.0 ± 0.7	−1.4 ± 0.9	4.2 ± 0.4	8.9 ± 1.3 *	30.8 ± 2.9 **
Malibatol B	0.0 ± 0.9	−0.8 ± 1.3	−6.1 ± 0.6	−10.9 ± 0.7	−17.0 ± 0.4
(+)-Parviflorol	0.0 ± 1.1	9.1 ± 2.5	21.2 ± 0.6 **	23.8 ± 1.0 **	−20.8 ± 0.2
*Cis*-resveratrol 10-*C*-Glc	0.0 ± 0.5	9.1 ± 2.0	20.9 ± 1.3 **	29.6 ± 2.6 **	33.6 ± 1.8 **
*Trans*-piceid	0.0 ± 3.3	1.3 ± 2.1	13.0 ± 2.6	19.9 ± 6.1 *	33.9 ± 3.6 **
1′*S*-Dihydrophayomphenol A_2_	0.0 ± 1.9	−0.8 ± 1.4	−1.2 ± 1.1	14.4 ± 0.5 **	38.0 ± 4.8 **
*Trans*-resveratrol	0.0 ± 1.7	8.5 ± 0.4	14.1 ± 0.9 **	37.5 ± 3.7 **	57.3 ± 2.5 **
Curcumin [22,23,25,27]	0.0 ± 3.7	0.1 ± 3.8	1.1 ± 2.2	−17.7 ± 1.3	−44.3 ± 0.3
Silybin [26,27,30,32]	0.0 ± 0.3	4.8 ± 1.1	7.7 ± 0.7	45.2 ± 8.8 **	77.0 ± 5.5 **

Each value represents the mean ± SEM (*n* = 4); asterisks denote significant differences from the control group, * *p* < 0.05, ** *p* < 0.01; commercial *trans*-resveratrol was purchased from Wako Pure Chemical Industries, Ltd. (Osaka, Japan), whereas silybin was from Funakoshi Co., Ltd. (Tokyo, Japan).

**Table 7:** Inhibitory effects of the constituents from bark of *Shorea roxburghiii* on LPS-activated NO production in mouse peritoneal macrophages.

Treatment	Inhibition (%)					IC_50_ (μM)
	0 μM	3 μM	10 μM	30 μM	100 μM	
(−)-Hopeaphenol (**1**)	0.0 ± 4.0 (100.0 ± 1.6)	42.3 ± 2.5 ** (118.9 ± 3.1)	64.9 ± 3.0 ** (119.1 ± 4.9)	73.9 ± 1.5 ** (130.6 ± 4.5)	80.5 ± 2.3 ** (111.5 ± 6.2)	4.6
(+)-Isohopeaphenol (**2**)	0.0 ± 2.9 (100.0 ± 3.5)	38.5 ± 4.6 ** (132.6 ± 1.1)	30.7 ± 4.0 ** (129.6 ± 5.6)	41.7 ± 6.3 ** (139.3 ± 4.4)	95.7 ± 1.0 ** (35.2 ± 1.5 ^#^)	38.5
Hemsleyanol D (**3**)	0.0 ± 3.8 (100.0 ± 2.5)	−4.3 ± 3.7 (120.1 ± 2.5)	8.4 ± 7.8 (135.3 ± 1.8)	34.8 ± 4.4 ** (93.3 ± 1.3)	-34.0 ± 1.8 (32.2 ± 2.9 ^#^)	
(−)-Ampelopsin H (**4**)	0.0 ± 5.4 (100.0 ± 2.4)	29.2 ± 1.8 ** (102.1 ± 1.0)	39.0 ± 2.8 ** (110.0 ± 4.0)	52.0 ± 3.9 ** (139.0 ± 0.3)	99.7 ± 0.5 ** (85.4 ± 0.9)	18.6
Vaticanol A (**5**)	0.0 ± 5.0 (100.0 ± 1.3)	30.3 ± 4.5 ** (122.1 ± 4.3)	32.2 ± 5.5 ** (132.2 ± 2.7)	14.5 ± 3.8 (138.4 ± 5.6)	0.5 ± 4.7 (136.2 ± 10.9)	
Vaticanol E (**6**)	0.0 ± 5.7 (100.0 ± 2.5)	-4.0 ± 8.9 (121.1 ± 2.9)	14.7 ± 5.9 (111.7 ± 7.2)	29.5 ± 5.3 ** (136.2 ± 4.0)	-21.7 ± 5.6 (122.1 ± 10.2)	
Vaticanol G (**7**)	0.0 ± 3.0 (100.0 ± 2.9)	8.2 ± 5.6 (130.2 ± 5.0)	25.1 ± 2.5 ** (120.3 ± 3.0)	26.7 ± 3.0 ** (123.3 ± 5.4)	44.1 ± 2.3 ** (131.6 ± 9.5)	
(+)-α-Viniferin (**8**)	0.0 ± 2.9 (100.0 ± 4.3)	27.8 ± 5.7 ** (123.4 ± 8.8)	46.3 ± 3.5 ** (98.8 ± 10.5)	75.4 ± 1.3 ** (87.7 ± 2.9)	97.9 ± 0.6 ** (36.5 ± 3.2 ^#^)	9.7
Pauciflorol A (**9**)	0.0 ± 4.1 (100.0 ± 4.5)	38.3 ± 3.8 ** (120.7 ± 5.6)	43.5 ± 3.2 ** (129.4 ± 3.8)	53.2 ± 2.2 ** (119.6 ± 2.2)	80.1 ± 1.4 ** (119.0 ± 2.4)	17.8
Hopeafuran (**10**)	0.0 ± 5.5 (100.0 ± 1.0)	30.6 ± 3.6 ** (125.7 ± 3.8)	33.3 ± 1.8 ** (128.7 ± 3.9)	33.6 ± 5.7 ** (128.9 ± 2.6)	67.7 ± 3.7 ** (117.5 ± 14.1)	45.9
(−)-Balanocarpol (**11**)	0.0 ± 7.2 (100.0 ± 6.5)	26.1 ± 7.6 ** (129.8 ± 7.4)	41.0 ± 5.2 ** (127.0 ± 4.8)	41.9 ± 5.2 ** (89.6 ± 4.8)	42.5 ± 2.7 ** (101.3 ± 2.9)	
(−)-Ampelopsin A (**12**)	0.0 ± 6.8 (100.0 ± 4.4)	16.5 ± 2.9 (127.3 ± 4.4)	23.1 ± 10.3 (139.0 ± 6.4)	32.0 ± 2.3 ** (128.3 ± 5.8)	32.9 ± 7.5 ** (116.3 ± 6.1)	
*Trans*-resveratrol 10-*C*-Glc (**13**)	0.0 ± 4.3 (100.0 ± 2.4)	39.0 ± 5.1 ** (126.6 ± 4.4)	43.6 ± 4.4 ** (125.2 ± 9.1)	38.2 ± 4.3 ** (119.8 ± 4.1)	45.4 ± 3.8 ** (113.0 ± 11.0)	
Phayomphenol A_1_ (**14**)	0.0 ± 4.9 (100.0 ± 2.0)	33.6 ± 6.0 ** (111.3 ± 1.1)	40.7 ± 6.5 ** (108.0 ± 1.9)	43.8 ± 4.2 ** (113.0 ± 2.1)	40.3 ± 3.8 ** (107.5 ± 5.3)	
Phayomphenol A_2_ (**15**)	0.0 ± 2.0 (100.0 ± 6.4)	35.2 ± 4.2 ** (102.0 ± 3.8)	38.1 ± 1.4 ** (102.9 ± 7.3)	42.4 ± 1.3 ** (120.9 ± 7.7)	45.4 ± 4.8 ** (104.7 ± 5.6)	
Vaticanol B	0.0 ± 5.4 (100.0 ± 2.0)	28.6 ± 2.6 ** (133.8 ± 2.7)	31.0 ± 4.8 ** (111.5 ± 7.1)	51.9 ± 1.7 ** (99.6 ± 5.1)	75.7 ± 1.0 ** (56.5 ± 2.8 ^#^)	26.8
Vaticanol C	0.0 ± 3.1 (100.0 ± 2.7)	35.4 ± 5.6 ** (124.4 ± 9.7)	45.3 ± 4.2 ** (105.4 ± 8.0)	59.2 ± 2.2 ** (94.2 ± 4.0)	96.5 ± 2.3 ** (11.8 ± 0.8 ^#^)	14.5
Malibatol A	0.0 ± 4.8 (100.0 ± 2.6)	28.5 ± 7.9 ** (133.8 ± 3.7)	42.5 ± 8.6 ** (131.6 ± 3.8)	47.8 ± 4.1 ** (106.3 ± 4.6)	77.3 ± 2.1 ** (96.0 ± 5.2)	23.0
Malibatol B	0.0 ± 5.7 (100.0 ± 41)	39.0 ± 5.7 ** (119.1 ± 3.4)	44.4 ± 4.8 ** (122.3 ± 2.3)	49.7 ± 2.3 ** (105.8 ± 7.0)	80.5 ± 3.3 ** (91.9 ± 4.6)	18.5
(+)-Parviflorol	0.0 ± 8.0 (100.0 ± 6.4)	34.8 ± 2.2 ** (122.8 ± 7.4)	48.6 ± 1.7 ** (130.1 ± 7.4)	49.7 ± 1.6 ** (92.7 ± 8.6)	50.9 ± 1.7 ** (95.7 ± 10.5)	40.9
*Cis*-resveratrol 10-*C*-Glc	0.0 ± 2.0 (100.0 ± 3.6)	26.5 ± 2.0 ** (102.9 ± 4.4)	35.5 ± 2.4 ** (109.6 ± 2.9)	47.0 ± 2.8 ** (99.6 ± 5.6)	46.5 ± 2.4 ** (91.8 ± 0.7)	
*Trans*-piceid	0.0 ± 3.5 (100.0 ± 1.8)	38.6 ± 3.1 ** (91.9 ± 7.7)	41.0 ± 2.7 ** (87.8 ± 5.5)	48.8 ± 2.1 ** (81.5 ± 3.3)	51.4 ± 5.3 ** (66.1 ± 4.8 ^#^)	59.8
1'*S*-Dihydrophayomphenol A_2_	0.0 ± 2.7 (100.0 ± 3.0)	40.1 ± 2.3 ** (117.3 ± 4.0)	40.6 ± 1.0 ** (115.6 ± 5.5)	48.6 ± 1.8 ** (109.9 ± 3.5)	49.4 ± 3.6 ** (113.5 ± 1.8)	
*Trans*-resveratrol	0.0 ± 2.6 (100.0 ± 1.4)	38.2 ± 6.3 ** (120.4 ± 1.7)	45.6 ± 1.3 ** (128.7 ± 3.6)	78.8 ± 1.1 ** (96.7 ± 10.1)	88.8 ± 1.0 ** (84.4 ± 3.0)	17.8
l-NMMA [26,27,30]	0.0 ± 3.1 (100.0 ± 0.9)	1.4 ± 2.8 (101.1 ± 5.7)	19.9 ± 2.8 ** (100.7 ± 6.2)	43.0 ± 2.1 ** (102.6 ± 4.2)	70.9 ± 1.6 ** (106.4 ± 4.6)	36.0
CAPE [26,27,30]	0.0 ± 2.1 (100.0 ± 1.5)	5.9 ± 5.2 (95.4 ± 0.7)	44.4 ± 3.2 ** (70.0 ± 4.0 ^#^)	86.2 ± 1.1 ** (71.4 ± 6.0 ^#^)	99.6 ± 0.1 ** (53.0 ± 1.4 ^#^)	11.0

Each value represents the mean ± SEM (*n* = 4); asterisks denote significant differences from the control group, * *p* < 0.05, ** *p* < 0.01; ^#^ cytotoxic effects were observed, and values in parentheses indicate cell viability (%) in MTT assay; commercial *trans*-resveratrol was purchased from Wako Pure Chemical Industries, Ltd. (Osaka, Japan), whereas L-NMMA and CAPE were from Sigma-Aldrich Chemical Co., LLC. (St. Louis, MO, USA).

**Table 8:** Inhibitory effects of the constituents from bark of *Shorea roxburghiii* on TNF-α-induced cytotoxicity in L929 cells.

Treatment	Inhibition (%)					IC_50_ (µM)
	0 µM	3 µM	10 µM	30 µM	100 µM	
(−)-Hopeaphenol (**1**)	0.0 ± 0.2	3.8 ± 1.5	28.9 ± 0.2 **	−1.8 ± 0.7	−6.7 ± 0.8	
(+)-Isohopeaphenol (**2**)	0.0 ± 0.5	−0.6 ± 1.1	0.3 ± 1.0	69.8 ± 1.4 **	−6.2 ± 0.5	
Hemsleyanol D (**3**)	0.0 ± 1.1	6.7 ± 1.8	17.6 ± 1.7 **	91.5 ± 2.1 **	10.0 ± 1.8	
(−)-Ampelopsin H (**4**)	0.0 ± 0.8	1.0 ± 2.1	−10.7 ± 0.3	−16.4 ± 0.4	−18.9 ± 0.3	
Vaticanol A (**5**)	0.0 ± 0.3	3.6 ± 0.4	4.0 ± 1.2	7.8 ± 1.5	28.9 ± 1.2 **	
Vaticanol E (**6**)	0.0 ± 0.4	6.7 ± 1.1	9.4 ± 0.6	3.7 ± 0.8	4.9 ± 0.5	
Vaticanol G (**7**)	0.0 ± 0.4	7.9 ± 0.8	9.7 ± 0.7	12.7 ± 0.6 *	57.6 ± 4.2 **	86.6
(+)-α-Viniferin (**8**)	0.0 ± 1.2	12.2 ± 1.2 *	38.3 ± 0.6 **	84.4 ± 2.5 **	78.5 ± 1.9 **	15.0
Pauciflorol A (**9**)	0.0 ± 1.0	8.2 ± 0.9	16.1 ± 0.7 **	54.6 ± 5.0 **	94.7 ± 4.6 **	26.7
Hopeafuran (**10**)	0.0 ± 0.7	8.3 ± 1.3	17.2 ± 1.3 **	66.4 ± 1.5 **	85.1 ± 1.2 **	22.0
(−)-Balanocarpol (**11**)	0.0 ± 1.1	4.5 ± 0.8	7.5 ± 1.7	10.4 ± 0.9 *	17.7 ± 3.2 **	
(−)-Ampelopsin A (**12**)	0.0 ± 1.1	4.1 ± 1.5	8.4 ± 1.1	14.6 ± 2.1 *	35.5 ± 1.5 **	
*Trans*-resveratrol 10-*C*-Glc (**13**)	0.0 ± 1.4	4.5 ± 2.2	4.4 ± 1.9	6.6 ± 1.6	6.6 ± 2.3	
Phayomphenol A_1_ (**14**)	0.0 ± 0.7	0.0 ± 0.6	1.8 ± 0.6	1.9 ± 0.6	1.5 ± 0.4	
Phayomphenol A_2_ (**15**)	0.0 ± 0.8	0.7 ± 0.7	1.4 ± 0.9	0.6 ± 0.5	−3.1 ± 0.7	
Vaticanol B	0.0 ± 0.7	0.0 ± 0.7	12.1 ± 1.1 *	7.9 ± 0.4	−9.8 ± 0.5	
Vaticanol C	0.0 ± 0.6	9.3 ± 0.3	14.4 ± 1.0 **	94.3 ± 2.6 **	−8.2 ± 0.2	18.2
Malibatol A	0.0 ± 0.5	8.1 ± 0.9	30.7 ± 3.8 **	86.9 ± 2.9 **	81.8 ± 4.3 **	12.3
Malibatol B	0.0 ± 1.2	16.8 ± 0.6 **	37.7 ± 3.3 **	90.7 ± 0.3 **	77.2 ± 2.9 **	10.2
(+)-Parviflorol	0.0 ± 0.8	4.5 ± 0.9	9.4 ± 2.2	13.0 ± 0.4 **	24.3 ± 0.9 **	
*Cis*-resveratrol 10-*C*-Glc	0.0 ± 0.7	4.8 ± 0.2	9.7 ± 1.2	14.5 ± 1.7 *	31.6 ± 1.5 **	
*Trans*-piceid	0.0 ± 0.9	3.4 ± 1.4	6.7 ± 2.6	3.6 ± 1.8	1.6 ± 1.7	
1′*S*-Dihydrophayomphenol A_2_	0.0 ± 0.3	2.0 ± 0.4	3.8 ± 0.5	2.5 ± 1.2	4.7 ± 0.5	
*Trans*-resveratrol	0.0 ± 1.4	1.4 ± 0.5	1.8 ± 0.8	2.1 ± 1.2	5.3 ± 0.6	
Silybin [27,36]	0.0 ± 2.6	5.3 ± 2.8	22.0 ± 3.8 **	48.0 ± 4.1 **	50.8 ± 3.9 **	60.4

Each value represents the mean ± SEM (*n* = 4); asterisks denote significant differences from the control group, * *p* < 0.05, ** *p* < 0.01.; commercial *trans*-resveratrol was purchased from Wako Pure Chemical Industries, Ltd. (Osaka, Japan), whereas silybin was from Funakoshi Co., Ltd. (Tokyo, Japan).

## Supplementary Materials

Supplementary materials can be found at www.mdpi.com/1422-0067/18/2/451/s1.
