# Supplementary material for: Quantitative Determination of Stilbenoids and Dihydroisocoumarins in Shorea roxburghii and Evaluation of Their Hepatoprotective Activity
Source: Int J Mol Sci. 2017 Feb 20;18(2):451. doi: 10.3390/ijms18020451 (PMC5343985; doi:10.3390/ijms18020451)
Supplement: Supplementary file 1 [file ijms-18-00451-s001.pdf]

Supplementary Materials: Quantitative Determination of Stilbenoids and Dihydroisocoumarins in *Shorea roxburghii* and Evaluation of their Hepatoprotective Activity

Kiyofumi Ninomiya, Saowanee Chaipech, Yusuke Kunikata, Ryohei Yagi, Yutana Pongpiriyadacha, Osamu Muraoka and Toshio Morikawa

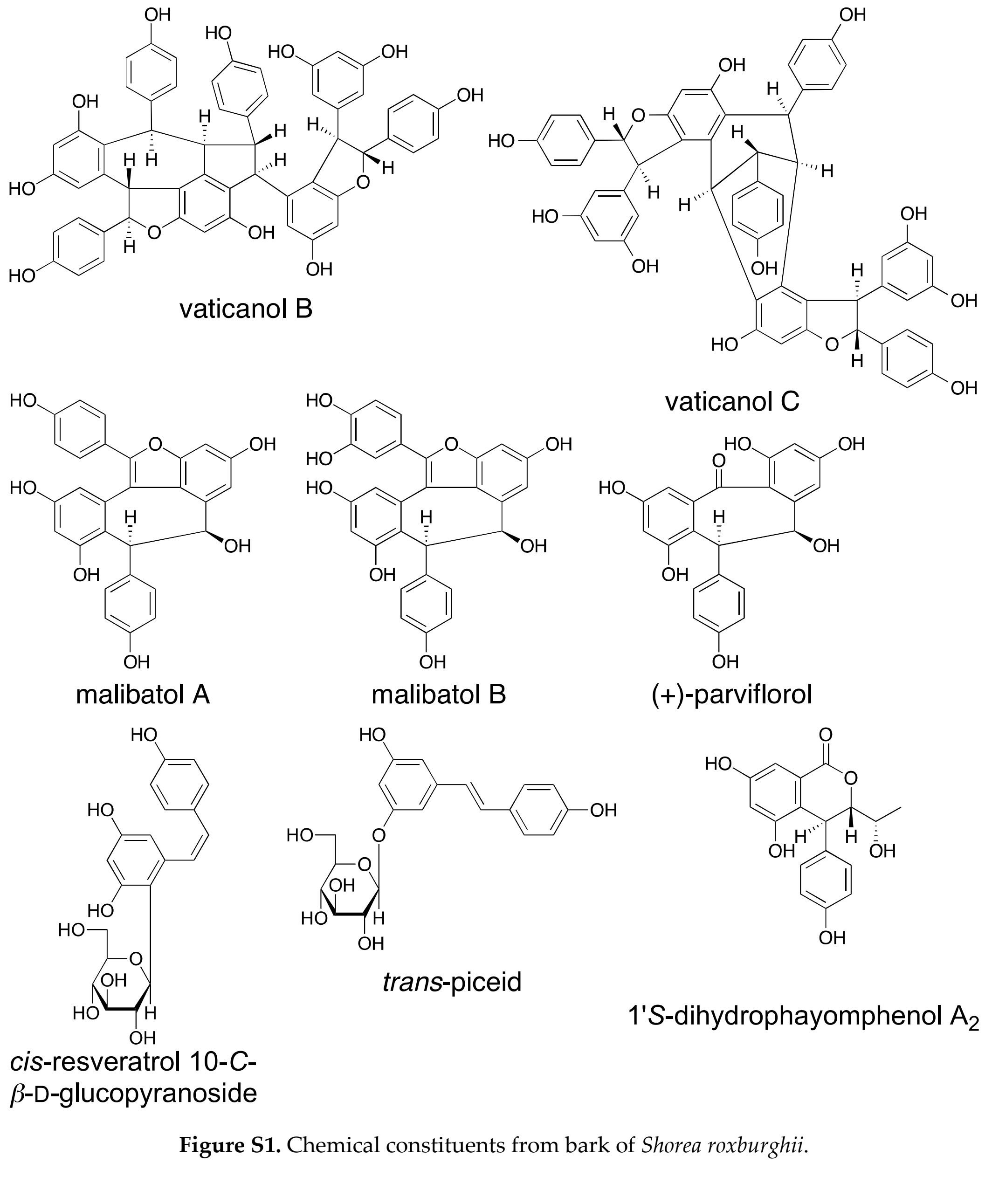

Figure S1. Chemical constituents from bark of *Shorea roxburghii*.

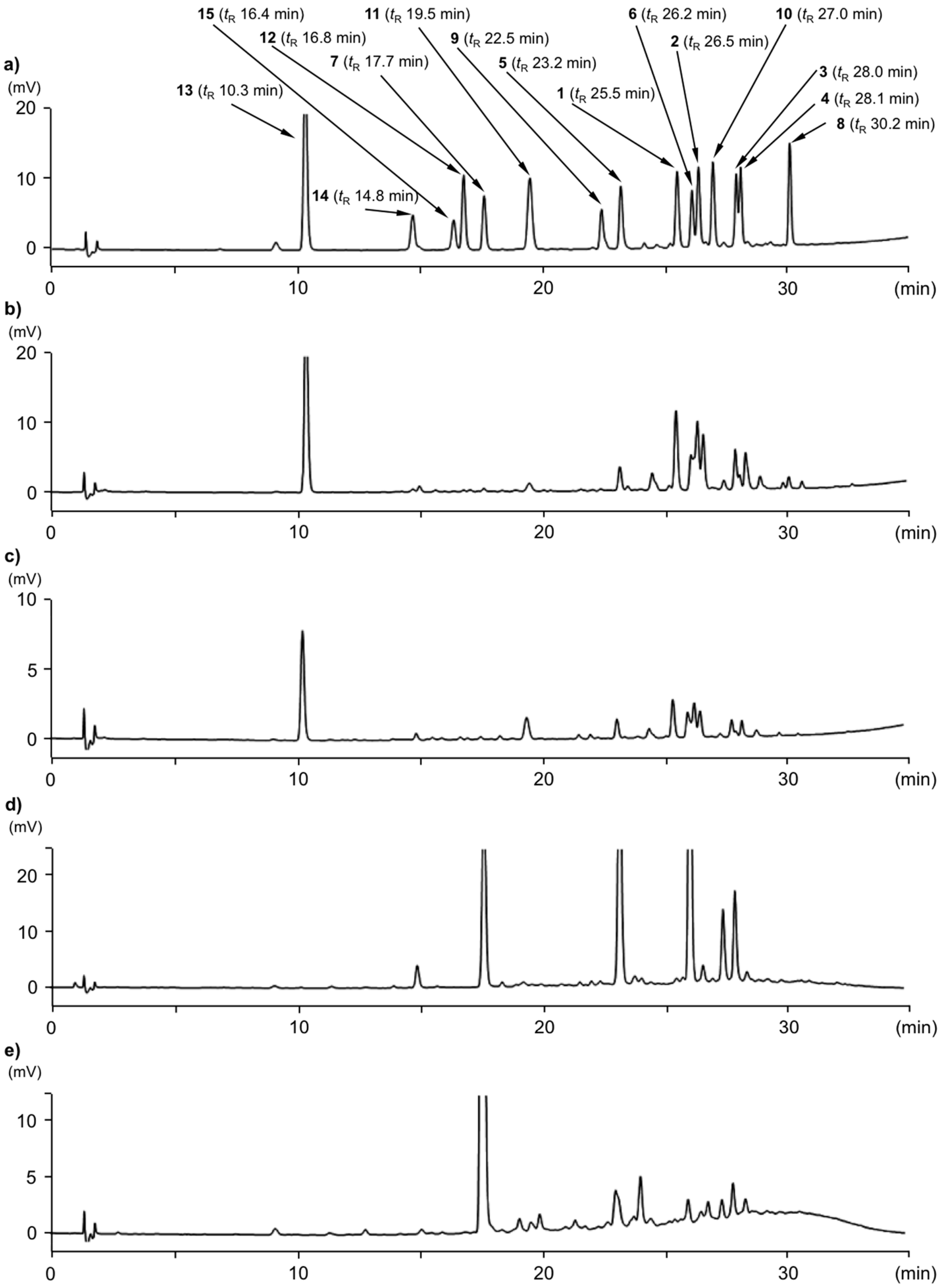

Figure S2. HPLC chromatograms (UV, 284 nm) of (**a**) standard solution mixture (each 25 µg/mL); methanol extracts from (**b**) bark of *Shorea roxburghii*; (**c**) wood of *Shorea roxburghii*; (**d**) bark of *Cotylelobium melanoxylon*; and (**e**) wood of *Cotylelobium melanoxylon*.
